# *Bothrops lanceolatus* Envenoming in Martinique: A Historical Perspective of the Clinical Effectiveness of Bothrofav Antivenom Treatment

**DOI:** 10.3390/toxins16030146

**Published:** 2024-03-13

**Authors:** Dabor Resiere, Jonathan Florentin, Hossein Mehdaoui, Hatem Kallel, Veronique Legris-Allusson, Papa Gueye, Remi Neviere

**Affiliations:** 1 Department of Critical Care Medicine, Toxicology and Emergency, CHU Martinique (University Hospital of Martinique), 97200 Fort-de-France, France; dabor.resiere@chu-martinique.fr (D.R.); jonathan.florentin@chu-martinique.fr (J.F.); hossein.mehdaoui@chu-martinique.fr (H.M.); papa.gueye@chu-martinique.fr (P.G.); 2 Cardiovascular Research Team UR5_3 PC2E Pathologie Cardiaque, Toxicité Environnementale et Envenimations, Université des Antilles, 97233 Fort-de-France, France; 3 Intensive Care Unit, Cayenne General Hospital, 97306 Cayenne, France; hatem.kallel@ch-cayenne.fr; 4 Department of Pharmacology, CHU Martinique (University Hospital of Martinique), 97200 Fort-de-France, France; veronique.legris-alluson@chu-martinique.fr

**Keywords:** snakebite, *Bothrops lanceolatus*, envenoming, Bothrofav, antivenom shortage, thrombosis, efficacy

## Abstract

Bothrofav, a monospecific antivenom, was introduced in June 1991 and has shown excellent effectiveness against life-threatening and thrombotic complications of *Bothrops lanceolatus* envenoming. Because of the reoccurrence of cerebral stroke events despite the timely administration of antivenom, new batches of Bothrofav were produced and introduced into clinical use in January 2011. This study’s aim was to evaluate the effectiveness of Bothrofav generations at treating *B. lanceolatus* envenoming. During the first period of the study (2000–2010), 107 patients were treated with vials of antivenom produced in June 1991, while 282 envenomed patients were treated with vials of antivenom produced in January 2011 in the second study period (2011–2023). Despite timely antivenom administration, thrombotic complications reoccurred after an interval free of thrombotic events, and a timeframe analysis suggested that the clinical efficacy of Bothrofav declined after it reached its 10-year shelf-life. In of the case of an antivenom shortage due to the absence of regular batch production, no adverse effects were identified before the antivenom reached its 10-year shelf-life, which is beyond the accepted shelf-life for a liquid-formulation antivenom. While our study does not support the use of expired antivenom for potent, life-threatening *B. lanceolatus* envenoming, it can be a scientific message to public entities proving the necessity of new antivenom production for *B. lanceolatus* envenoming.

## 1. Introduction

Features of human *Bothrops* spp. snake envenomation include marked local damage which may be associated in some severe cases with abnormal hemostasis with systemic hemorrhage. Coagulotoxicity has been attributed to the synergistic action of several venom toxins [1,2,3]. The most toxic constituents of *Bothrops* spp. venom involved in impaired hemostasis and hemorrhage are snake venom serine proteases (SVSPs), snake venom metalloproteinases (SVMPs), phospholipase A2 (PLA2), disintegrins (DISs), and C-type lectin-like proteins (CTLs) [1,4]. While islands of the Caribbean Sea are habitats for many animal species, including dozen of *Bothrops* spp. snake species, *B. lanceolatus* is the only venomous pit viper species endemic to Martinique, a French Caribbean Island of the Lesser Antilles. The protein composition of *B. lanceolatus* venom harvested from snakes maintained in captivity has been investigated using a snake venomics approach, including the fractionation of crude venoms using reverse-phase high-performance liquid chromatography HPLC and the analysis of each chromatographic fraction. Overall, the protein composition of freeze-dried *B. lanceolatus* venom harvested from snakes maintained in captivity showed that SVMPs were the predominant family, ranging from 41–74% of *B. lanceolatus* proteins, followed by SVSPs (11–14%), PLA2s (9–16%), and L-amino acid oxidase (~3%) [5,6].

In Martinique, *B. lanceolatus* is responsible for approximately 15–25 cases of envenoming every year [7,8]. A *B. lanceolatus* snakebite induces typical local damage similar to that described after *Bothrops* spp. snakebites, including edema, blistering, dermonecrosis, myonecrosis and hemorrhage. In sharp contrast with the hemorrhagic pattern of *Bothrops* spp. snake envenoming, *B. lanceolatus* snakebites are infrequently responsible for systemic bleeding and anticoagulability. Instead, *B. lanceolatus* envenoming is characterized by a unique pathophysiological pattern resulting in severe thrombotic events within 48 h of the bite if no rapid antivenom administration is initiated [9,10,11]. Before the era of immunotherapy, *B. lanceolatus* envenoming was associated with systemic arterial thrombotic complications leading to cerebral, myocardial, or pulmonary infarctions in 25–30% of cases and was fatal in about 10% of cases [8,9,10,11,12]. The mechanisms behind the thrombotic effect induced by *B. lanceolatus* venom are largely unknown. Proposed mechanisms of these thrombotic events include direct injury to the vascular endothelium by venom toxins and vascular endothelial cell activation related to inflammatory processes [2,13,14]. Notably, the proteomic and toxicological profiles of *B. lanceolatus* venom are similar to those of other *Bothrops* sp. snakes [5,6,15].

Owing to the potential severity of *B. lanceolatus* envenoming, the monospecific antivenom Bothrofav was introduced in the early 1990s for the prevention of the development of thrombotic complications and other manifestations of *B. lanceolatus* envenoming [10,11,12]. Bothrofav is an F(ab’)2 immunoglobulin preparation of equine origin, initially manufactured by Sanofi-Pasteur, Paris. When administered in a timely manner, Bothrofav has demonstrated excellent safety and effectiveness in reducing mortality to zero and preventing thrombosis complications [11,16,17]. Preclinical studies confirmed the efficacy of this antivenom at neutralizing lethal and other toxic activities of *B. lanceolatus* venom [18,19]. Third-generation antivenomics previously demonstrated the immunological reactivity of Bothrofav when confronted with the homologous venom of *B. lanceolatus*. The antivenom was able to immunocapture all protein venom fractions with the only exception of the first HPLC peaks which correspond to low-molecular-mass peptides, which may be non-immunogenic [5]. These antivenomics observations correlate well with the high capacity of Bothrofav to neutralize lethal, local, and systemic hemorrhagic and the edema-forming, thrombocytopenic, phospholipase A2, and proteinase activities of *B. lanceolatus* venom [5,15,20].

The first generation of antivenom was used until the late 2010s, and a reoccurrence of cerebral stroke events was reported in August 2003 [8,9,10]. In the absence of the regular production of antivenom vials, the reduction in immunotherapy effectiveness was attributed to a drop in the neutralizing potency of the antivenom [10] since liquid antivenoms are known to lose efficacy several years after their manufacture; in this case, over a decade [21]. A new generation of Bothrofav antivenom was manufactured in 2010 and introduced into clinical use in January 2011. Both Bothrofav generations were produced using identical manufacturing methods by the same manufacturer (Sanofi-Pasteur, Lyon, France). Compared with the first antivenom batch, which was prepared from a venom pool of only seven adult snakes, the second-generation Bothrofav batch was prepared from a venom pool of twenty-seven snakes, including males and females as well as juveniles, collected from various regions of Martinique, covering the entire geographical distribution of *B. lanceolatus*. This new batch showed a high neutralizing efficacy in preclinical tests, including the neutralization of toxic activities in vitro and in antivenomics tests [15,20]. As for the case of the first Bothrofav generation, no regular production of new antivenom vials was anticipated. In addition, the safety and effectiveness of the second generation of Bothrofav in treating *B. lanceolatus* envenoming has not been previously reported.

The primary objective of the present study was to evaluate the safety and effectiveness of new Bothrofav batches introduced into clinical use in January 2011 in comparison with Bothrofav batches manufactured in 1991 to treat *B. lanceolatus* envenoming. The primary endpoint of the study was the length of hospital stay. The second objective was to analyze the effects of Bothrofav vial storage duration on the occurrence of cerebral stroke. The secondary endpoint was the number of cerebral strokes occurring after the timely administration of Bothrofav.

## 2. Results

A total of 389 patients treated with a standardized antivenom protocol were included in this study from January 2000 to March 2023. During the first study period (January 2000–December 2010), 107 patients envenomed by *B. lanceolatus* were treated with batches of Bothrofav produced in June 1991 (Bothrofav#1). A renewed Bothrofav preparation was available in January 2011 (Bothrofav#2) and has been used since then. During the second study period (January 2011–March 2023), 282 envenomed patients were treated with new batches of Bothrofav (Bothrofav#2). Overall, both generations of Bothrofav have been manufactured without the further production of new batches. Hence, the age of the Bothrofav batches at the time of admission only depend on their date of manufacture and commercialization, i.e., June 1991 for Bothrofav#1 and January 2011 for Bothrofav#2.

Despite some missing patient registry data, critical information such as clinical and biological presentation, envenoming grade severity, timing of antivenom administration, antivenom protocol, thrombotic complications, and follow-up were fully available for all envenomed patients. Table 1 summarizes the main characteristics of the 389 envenomed patients. Patients treated in the first period (2000–2010) with Bothrofav #1 had higher-grade severity scores compared with patients treated in the second period (2011–2023) with Bothrofav #2. The time from snakebite to antivenom administration was similar, with a median of 3:30 h (IRQ 2:30–7:00) for Bothrofav #1 and 4:53 h (IRQ 3:45–7:33) for Bothrofav #2. Premedication drugs given to patients before antivenom administration by dedicated physicians included combinations of antihistamines and/or corticosteroids. Early mild and moderate adverse reactions occurred in less than 10%, with no difference between the Bothrofav #1 and Bothrofav #2 treatment groups. There was no serum sickness diagnosed in this series of patients.

Notably, abnormal neurologic presentation at admission, such as cerebral stroke prior to antivenom administration, was more frequent in the 2000–2010 period compared with the 2011–2023 period. Despite similar clinical management approaches, the hospital stay was longer in patients attended to during the 2000–2010 period compared with patients attended to during the 2010–2023 period (Table 2). These results suggest that the overall *B. lanceolatus* envenoming severity was more severe in the first compared with the second study period.

**Figure 1:**
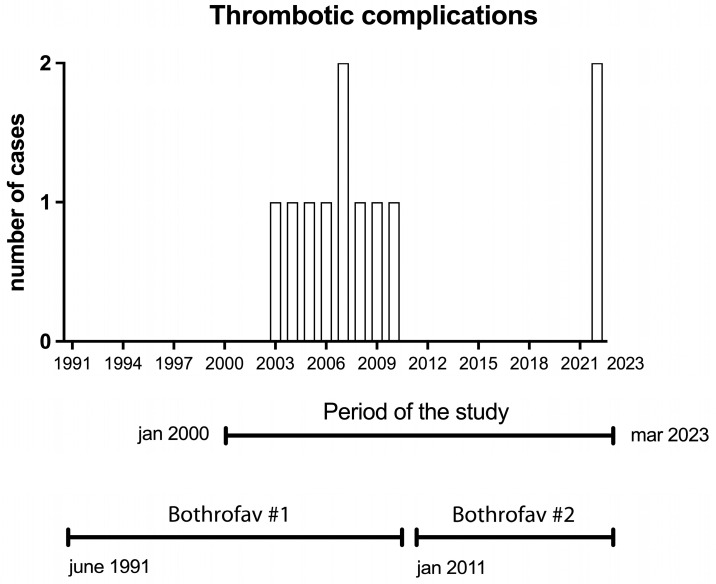
Absolute number of cerebral thrombotic events associated with timely treatment of *B. lanceolatus* envenoming using Bothrofav according to protocol. Timeline is presented as 3-year tick intervals.

The absolute number of cerebral thrombotic events over years that occurred at least 6 h after Bothrofav administration is displayed Figure 1. The timeline is presented as a 3-year tick interval, which is the WHO shelf-life for liquid-formulation antivenom. Following the commercialization of the first batch of antivenom, Bothrofav (Bothrofav #1), in June 1991, the first cerebral stroke occurring after adequate and timely antivenom treatment was recorded in August 2003. Nine cerebral stroke events were observed between August 2003 and December 2010 (Figure 1). Following the commercialization of the new Bothrofav batch (Bothrofav #2) in January 2011, no cerebral stroke events occurred after adequate and timely antivenom treatment until February 2022. While there were no cerebral stroke events in 2023, two stroke events were observed in 2022.

A timeframe analysis suggests that cerebral stroke did not occur episodically but rather after an interval free of events of 10–12 years. This observation suggests that the clinical effectiveness of both Bothrofav generations declined after the 10-year antivenom shelf-life. The absolute number of cerebral strokes occurring after timely antivenom treatment was higher in the 2000–2010 period when compared with the 2011–2023 period due to the longer time of Bothrofav#1 use compared with the length of time of Bothrofav#2 use.

## 3. Discussion

The present study investigated the efficacy of Bothrofav antivenom in patients with *B. lanceolatus* envenoming presenting at the University Hospital of Martinique from January 2000 to March 2023. During this period, two successive Bothrofav batches manufactured in June 1991 (Bothrofav#1) and in January 2011 (Bothrofav#2) were used from 2000 to 2010 and from 2011 to 2023, respectively. We found that both Bothrofav batches proved to be highly effective at controlling systemic manifestations of *B. lanceolatus* envenoming when used within their regular shelf-life period. Our results also suggest that the antivenom’s clinical efficacy at preventing cerebral thrombotic complications declined after it reached its 10-year shelf-life, whichever Bothrofav batch was used. Indeed, as it has a liquid formulation, the expiration date of Bothrofav antivenom is usually set to three years after the production date, even if adequate efficacy of expired and aged antivenoms can be documented many years after their production [21,22,23,24].

Potential reasons for the failure of antivenom administration in human snakebite envenoming are related to insufficient pools of captive and wild snakes used to immunize horses for antivenom production, the inability of antibodies to bind the venom toxins, prior irreversible damaging effects of venom, the inability of antivenom to reach the venom target, and inadequate venom and antivenom pharmacokinetics. Another possible mechanism for antivenom treatment failure is the decline in its neutralizing potency over time. The expiration date of lyophilized snake antivenom is usually set to 5 years after its production date. Liquid-formulation antivenom has a shelf-life of 3 years even when stored at 2–8 °C [21]. In some cases, this initial shelf-life has subsequently been extended thanks to preclinical lab tests confirming sustained efficacy many years after the production date [21,22,23,24,25]. In our observational study, a major concern was the requirement to use aged Bothrofav vials, sometimes far beyond their expiration date, for the clinical management of *B. lanceolatus* envenoming. While experience with using expired antivenoms has been reported in low-income countries, the shortage of Bothrofav cannot be attributed to financial constraints for patients or whole communities in Martinique, where out-of-pocket payments for medical services do not prevail. Indeed, Martinique, an overseas department of France and part of the European Union, is one of the most highly developed islands in the Caribbean basin, classified as high (41st) in terms of global human development at the world level. Instead, our study identified the insufficient production capacity of the only available antivenom against *B. lanceolatus* envenoming as the main cause leading to the shortage in and administration of expired antivenom for the treatment of *B. lanceolatus* envenoming. In this context, it should be noted that the French National Health authorities have restricted the administration of the monospecific Bothrofav antivenom to *B. lanceolatus* envenoming (15–25 cases per year at most), which placed the pharmaceutical industry under de facto financial pressure regarding such a small market size and the high production cost and short shelf-life of Bothrofav. Both generations of Bothrofav were produced using identical manufacturing processes by the same pharmaceutical company (Sanofi-Pasteur, Lyon, France). Compared with the first-generation batches of antivenom, which were prepared from a venom pool of only seven adult snakes, the second-generation Bothrofav batches were prepared from a venom pool of twenty-seven snakes, including males and females as well as juveniles, collected from various regions of Martinique, covering the entire geographical distribution of *B. lanceolatus*. Moreover, the vaccine division of Sanofi-Pasteur (Sanofi-Pasteur, Lyon, France), the only company manufacturing Bothrofav, has progressively reduced its antivenom production facilities and signed an agreement at the end of December 2017 to divert Bothrofav manufacturing to MicroPharm Ltd., which also resulted in a production delay.

During the first period of the study, when the Bothrofav batches manufactured in June 1991 were used, cerebral strokes occurring after adequate and timely antivenom treatment could be related to high envenoming severity rather than reduced toxin neutralization by Bothrofav. Envenoming severity was supported by 32% of patients with grade 3 and 4 severity scores and their related lengths of hospital stay, i.e., 4.5 days and 12.2 days, respectively. This interpretation is not tenable since the timeframe analysis suggests that cerebral stroke events did not occur episodically but rather after an interval free of events of 10–12 years. In addition, during the second period of the study, when the Bothrofav batches manufactured in 2011 were used, cerebral stroke events also occurred after an interval free of events of 10–12 years. Indeed, the new batches of Bothrofav produced in January 2011, using a large pool of venoms from male and female snakes, demonstrated full efficacy against systemic envenoming and prevented the development of thrombotic complications, but two new cerebral strokes occurred in March 2023, suggesting that the neutralization capacity of the second Bothrofav batch also declined after a 10-year period. Considering that this is a retrospective study over 20 years old, any conclusion regarding the outcomes of the two treated groups can be drawn. Factors that may influence the issue of antivenom ineffectiveness on clinical/laboratory parameters include changes in the clinical management of patients over time and changes in the processes of producing Bothrofav and evaluation criteria for thrombotic disorders over the years. Overall, these factors can interfere with the results over the years, which reduces the study’s counterpart regarding causality.

An alternative explanation for Bothrofav antivenom failure may be related to changes in *B. lanceolatus* venom characteristics over time. A high degree of venom variation is observed at the intraspecific level in different situations represented by ontogenetic variations related to sex or according to geographic distribution [26]. Environmental factors such as climate, seasonal variation, and snake diet can also influence the relative abundances of different classes of toxins in adults [25,26,27,28]. Likewise, hibernation, the recency of last venom extraction, and temperature participate in the great variability in venom composition within a population [25,26,27,28]. Consequently, for human envenoming, venom variation may result in significant differences in symptoms arising from snake bites and innate mechanisms initiating inflammatory reactions critical to host protection and can also undermine the efficacy of the antivenom, which may have a reduced potential to neutralize such a variety of different toxins [29,30,31]. Although venom variation and the host response might have been different during the two periods of Bothrofav use, it is very unlikely that these changes explain the periods free of complications following Bothrofav’s initial production and renewal. Notably, there is no report available studying *B. lanceolatus* venom variability.

## 4. Conclusions

In conclusion, the observational data presented here indicate that the clinical efficacy of Bothrofav in the prevention of thrombotic complications declined after it reached its 10-year shelf-life. In conditions of antivenom shortage, aged batches of liquid antivenom up to 10 years of age should not be used in *B. lanceolatus* envenoming. Our findings call for further studies on the stability of antivenoms beyond their shelf-lives in order to take advantage of their stability while paying close attention to the decline in their neutralizing activity so as to avoid complications such as the ones described in this study.

Regarding the use of expired antivenom for potent, life-threatening *B. lanceolatus* envenoming, we acknowledge the ethical issue with a study of such a sensitive topic. We also stress that the study does not endorse or support the use of expired antivenoms. Actually, the study can be a scientific message to public entities, proving the necessity of new antivenom production for *B. lanceolatus* envenoming.

## 5. Materials and Methods

**Ethics and retrospective chart review:**

Information regarding antivenom treatment was provided to each patient. An explanation of risks and descriptions of the different procedures, including Bothrofav administration, were provided to the patients. This study was conducted in accordance with the Declaration of Helsinki and approved by the Institutional Review Board of CHU Martinique Hospital (IRB #01072019). Bothrofav has a Nominative Temporary Authorization for use in France (Product ID Code: 3400958910388) granted by the French National Security of Health Agency (ANSM). In cases requiring expired Bothrofav vials, the decision of Bothrofav administration was left to the clinician’s discretion.

**Participants:**

Patients with a diagnosis of *B. lanceolatus* envenoming presenting at the University Hospital of Martinique were retrospectively studied from January 2000 to March 2023. A historical comparator group of patients treated with Bothrofav produced in the early 1990s (Bothrofav#1) served as controls (2000–2010) for the evaluation of Bothrofav#2’s effectiveness against *B. lanceolatus* envenoming (2011–2023). During both phases of this study, all patients bitten by *B. lanceolatus* were admitted to the emergency department at the University Hospital of Martinique (where antivenom is available). Antivenom administration and intensive supportive care strictly followed the same standardized protocol through the entire study period (2000–2023).

**Data collection:**

An electronic system (DXCARE, Dedalus, France) was used to record information regarding each patient’s detailed history, physical examination data, and other relevant data related to snakebite envenomation with the help of a dedicated form. Clinical examinations were performed by senior physicians (an emergency physician, intensivist, and an anesthesiologist). Paper copies of the medical data were referenced, and the variables of interest were predefined in the study protocol written by medical staff. Epidemiological data were collected and processed using Microsoft Excel software (Excel 2013 version 15.0), Study endpoints were selected to demonstrate the clearest possible difference between treatment groups while minimizing confounders. Results were summarized, and endpoints were directly compared between groups.

**Diagnosis and antivenom therapy for snakebite envenoming:**

When seen, the culprit snake was identified based on the patient’s description, photographs, or a physical examination of the captured snake. Snakebite was confirmed via the observation of two puncture wounds resulting from snake fang marks. Epidemiological and clinical data, including the age and gender of patients, the date and time of the bite, the anatomical site of the bite, the grade of severity of envenoming, clinical manifestations and laboratory tests at admission, the date and time of hospital admission, and antivenom administration were collected from the medical records of the patients. The development of envenoming, occurrence of adverse events, duration of hospital stays, and outcome were monitored for each patient. We excluded patients hospitalized for snakebite envenoming lasting more than 48 h after the bite, those who did not receive antivenom, and those who did not present clinical or biological signs of envenoming. Upon admission, all patients were checked for their tetanus vaccination status and underwent a quick tetanus test. Anti-tetanus prophylaxis was administered accordingly.

The severity of envenoming of each patient was measured according to a previously defined clinical severity scale [8]. This grading system is based on four grades (I: mild; II: moderate; III: severe; and IV: major), including a large number of signs and symptoms associated with a *B. lanceolatus* bite (evidence of fang marks) [8]. Severity grade scores were as follows: score I, mild—no pain and no general signs; score II, moderate—local swelling confined to two segments of the bitten limb; score III, severe—moderate pain, regional oedema, the extension of swelling beyond two segments, persistent and resistant pain to analgesics, and no general signs; and score IV, major—swelling spreading to the trunk, persistent and resistant pain to analgesics, general signs (vomiting, headache, and abdominal or chest pain), hypotension, isolated thrombocytopenia, and disseminated intravascular coagulation.

Envenomed patients were treated using an infusion of the antivenom Bothrofav at doses adjusted to the severity grade of the case [8,16]. Bothrofav antivenom was given via intravenous infusion or by electrical syringe, diluted in isotonic saline with a flow of about 10 to 20 mL/h. Patients with score I, mild, received no antivenom therapy. Patients with moderate, severe, and major score severities received 40 mL, 60 mL, and 80 mL, respectively. A potential allergic reaction was considered in all patients. Undesirable early adverse reactions that may occur with this equine antivenom including mild (skin and subcutaneous tissue changes, such as generalized erythema, rash, periorbital edema, or angioedema), moderate (features suggesting respiratory, cardiovascular, or gastrointestinal involvement, including dyspnea, stridor, wheezing, nausea, vomiting, dizziness, diaphoresis, chest or throat tightness, and abdominal pain), and severe (hypoxia, hypotension, or neurological compromises, including cyanosis, oxygen saturation < 90%, confusion, collapse, and loss of consciousness) anaphylactoid/anaphylactic reactions were recorded [31].

**Statistical analysis:**

Analyses were performed using Excel (Microsoft Office 2019) and SPSS Statistics version 18 (IBM SPSS, Inc., Chicago, IL, USA) for Windows. Results are reported as the number of patients in whom the data were recorded, as median and inter-quartile range (IQR) values, or as absolute numbers of cases and percentages. Time is expressed as hours and minutes (hh:mm). Categorical variables were presented as absolute values and percentages and compared using Fisher’s exact test. Continuous variables were tested for normality using the Kolmogorov–Smirnov test and Shapiro–Wilk tests. Variables with normal distribution were compared using an independent-samples two-tailed t-test. Variables with non-normal distribution were compared using the Mann–Whitney U-test. The level of statistical significance was set at *p*-value < 0.05.

## Figures and Tables

**Table 1:** Characteristics of patients bitten by *B. lanceolatus* treated with Bothrofav.

	Bothrofav#1 Period 2000–2010 (107 Patients)	Bothrofav#2 Period 2011–2023 (282 Patients)	*p*
**Age** (years)	47 ± 17	46 ± 17	0.798
**Gender** (Male)	84 (78.5%)	205 (72.7%)	0.299
**Medical history**			
Cardiovascular risk	17 (15.9%)	49 (17.4%)	0.765
Coagulopathy/anticoagulant	0 (0%)	4 (1.4%)	0.579
**Envenoming presentation**			
Time of bite (hh:mm)	2:00 p.m. (9:30 a.m.–7:00 p.m.)	2:00 p.m. (9:00 a.m.–7:00 p.m.)	0.710
Time of hospital admission (hh:mm)	7:30 p.m. (1:00 p.m.–12:00 p.m.)	5:00 p.m. (11:05 a.m.–10:00 p.m.)	0.708
Time from bite to hospitalization (hours)	2:30 (1:30–6:00)	3:50 (2:47–6:33)	0.161
Snake identified	39 (36.4%)	85 (30.1%)	0.273
Anatomic site of bite			
- Upper limb	46 (43.0%)	116 (41.1%)	0.818
- Lower limb	60 (56.1%)	165 (58.5%)	0.730
**Grade of envenoming**			
Grade 1	0 (0%)	0 (0%)	
Grade 2	73 (68.2%)	168 (59.6%)	0.129
Grade 3	14 (13.1%)	44 (15.6%)	0.633
Grade 4	20 (18.7%)	21 (7.4%)	**0.003**
**Clinical presentation at admission**			
Heart rate > 100 bpm	17 (15.9%)	37 (13.1%)	0.512
Circulatory shock	1 (0.9%)	2 (0.7%)	>0.999
Confusion/headache/dizziness	9 (8.4%)	24 (8.5%)	>0.999
Stroke before Bothrofav treatment	5 (4.7%)	0 (0%)	**0.002**
Individual NIHSS score	1, 8, 12, 13, 20	NA	-
**Biological analysis at admission**			
Normal platelet count and prothrombin time	87 (81.3%°)	232 (82.2%)	0.883
Thrombocytopenia	15 (14.0%)	27 (9.7%)	0.206
Disseminated intravascular coagulation	3 (2.8%)	6 (2.1%)	>0.999
**Clinical evolution**			
Anaphylaxis reactions (Bothrofav)	3 (2.8%)	6 (2.1%)	>0.999
Thrombocytopenia	7 (6.5%)	26 (9.2%)	0.541
Stroke after Bothrofav treatment	5 (4.7%)	2 (0.7%)	**0.019**
Individual NIHSS score	(1, 1, 4, 8, 15)	(1, 4)	-
**Neuroimaging procedures**			
Number of brain MRI exams	15 (14.0%)	27 (9.6%)	0.206
Abnormal brain MRI exams	10 (9.3%)	2 (0.7%)	**<0.001**

Results are reported as mean ± SD values, median and inter-quartile ranges (IQRs) or as absolute numbers of cases and percentages. Abbreviation: NIHSS: National Institutes of Health Stroke Scale; MRI: magnetic resonance imaging. Tachycardia was defined as a heart rate of up to 100 beats per minute. Thrombocytopenia was defined as a platelet count of less than 150 Giga/L.

**Table 2:** Management and outcome of patients envenomed by *B. lanceolatus* and treated with Bothrofav.

	Bothrofav#1 Period 2000–2010 (107 Patients)	Bothrofav#2 Period 2011–2023 (282 Patients)	*p*
Time from the bite to antivenom (hh:mm)	3:30 (2:30–7:00)	4:53 (3:53–7:33)	0.133
Antivenom in less than 6 h	79 (73.8%)	194 (68.8%)	0.385
Documented soft tissue infection	6 (5.6%)	24 (8.5%)	0.401
Antibiotics	34 (31.8%)	65 (23.0%)	0.090
Length of hospital stay (days)	2.5 (1–3.5)	1 (1–2.0)	0.001
Death	1 (0.9%)	0 (0%)	0.275

Results are reported as median and inter-quartile range (IQRs) or as absolute numbers of cases and percentages.

## Data Availability

All relevant data are contained within the manuscript.

## References

[1] Gutierrez J.M., Calvete J.J., Habib A.G., Harrison R.A., Williams D.J., Warrell D.A. (2017). Snakebite envenoming. Nat. Rev. Dis. Primers.

[2] Cavalcante J.S., de Almeida D.E.G., Santos-Filho N.A., Sartim M.A., Baldo A.d.A., Brasileiro L., Albuquerque P.L., Oliveira S.S., Sachett J.A.G., Monteiro W.M. (2023). Crosstalk of inflammation and coagulation in *Bothrops* snakebite envenoming: Endogenous signaling pathways and pathophysiology. Int. J. Mol. Sci..

[3] Moore G.W. (2022). Snake venoms in diagnostic hemostasis and thrombosis. Semin. Thromb. Hemost..

[4] Monteiro W.M., Contreras-Bernal J.C., Bisneto P.F., Sachett J., Da Silva I.M., Lacerda M., Da Costa A.G., Val F., Brasileiro L., Sartim M.A. (2020). *Bothrops atrox*, the most important snake involved in human envenomings in the Amazon: How venomics contributes to the knowledge of snake biology and clinical toxinology. Toxicon X.

[5] Gutiérrez J.M., Sanz L., Escolano J., Fernández J., Lomonte B., Angulo Y., Rucavado A., Warrell D.A., Calvete J.J. (2008). Snake venomics of the Lesser Antillean pit vipers *Bothrops caribbaeus* and *Bothrops lanceolatus*: Correlation with toxicological activities and immunoreactivity of a het-erologous antivenom. J. Proteome Res..

[6] Larréché S., Bousquet A., Chevillard L., Gahoual R., Jourdi G., Dupart A.-L., Bachelot-Loza C., Gaussem P., Siguret V., Chippaux J.-P. (2023). *Bothrops atrox* and *Bothrops lanceolatus* venoms in vitro Investigation: Composition, procoagulant effects, co-factor dependency, and correction using antivenoms. Toxins.

[7] Campbell J.A., Lamar W.W. (1990). The Venomous Reptiles of Latin America.

[8] Resiere D., Megarbane B., Valentino R., Mehdaoui H., Thomas L. (2010). *Bothrops lanceolatus* bites: Guidelines for severity assessment and emergent management. Toxins.

[9] Malbranque S., Piercecchi-Marti M.D., Thomas L., Barbey C., Courcier D., Bucher B., Richard A., Smadja D., Warrel D.A. (2008). Fatal diffuse thrombotic microangiopathy after a bite by the “Fer-de-Lance” pit viper (*Bothrops lanceolatus*) of Martinique. Am. J. Trop. Med. Hyg..

[10] Thomas L., Chausson N., Uzan J., Kaidomar S., Vignes R., Plumelle Y., Bucher B., Smadja D. (2006). Thrombotic stroke following snake bites by the “Fer-de-Lance” *Bothrops lanceolatus* in Martinique despite antivenom treatment: A report of three recent cases. Toxicon.

[11] Thomas L., Tyburn B., Bucher B., Plumelle Y., Ketterle J., Pecout F., Rieux D., Smadja D., Garnier D. (1995). Prevention of thromboses in human patients with *Bothrops lanceolatus* envenoming in Martinique: Failure of anticoagulants and efficacy of a monospecific antivenom. Research Group on Snake Bites in Martinique. Am. J. Trop. Med. Hyg..

[12] Merle H., Donnio A., Ayeboua L., Plumelle Y., Smadja D., Thomas L. (2005). Occipital infarction revealed by quadranopsia following snakebite by *Bothrops lanceolatus*. Am. J. Trop. Med. Hyg..

[13] Silva de França F., Gabrili J.J.M., Mathieu L., Burgher F., Blomet J., Tambourgi D.V. (2021). *Bothrops lanceolatus* snake (Fer-de-lance) venom triggers inflammatory mediators’ storm in human blood. Arch. Toxicol..

[14] Delafontaine M., Villas-Boas I.M., Pidde G., van den Berg C.W., Mathieu L., Blomet J., Tambourgi D.V. (2018). Venom from *Bothrops lanceolatus*, a Snake Species Native to Martinique, Potently Activates the Complement System. J. Immunol. Res..

[15] Pla D., Rodriguez Y., Resiere D., Mehdaoui H., Gutierrez J.M., Calvete J.J. (2019). Third-generation antivenomics analysis of the preclinical efficacy of Bothrofav antivenom towards *Bothrops lanceolatus* venom. Toxicon X.

[16] Thomas L., Tyburn B., Lang J., Ketterle J. (1996). Early infusion of a purified monospecific F(ab’)2 antivenom serum for *Bothrops lanceolatus* bites in Martinique. Lancet.

[17] Thomas L., Tyburn B., Ketterlé J., Biao T., Mehdaoui H., Moravie V., Rouvel C., Plumelle Y., Bucher B., Canonge D. (1998). Prognostic significance of clinical grading of patients envenomed by *Bothrops lanceolatus* in Martinique. Members of the research group on snake bite in Martinique. Trans. R. Soc. Trop. Med. Hyg..

[18] Bucher B., Canonge D., Thomas L., Tyburn B., Robbe-Vincent A., Choumet V., Bon C., Ketterlé J., Lang J. (1997). Clinical indicators of envenoming and serum levels of venom antigens in patients bitten by *Bothrops lanceolatus* in Martinique. Research group on snake bites in Martinique. Trans. R. Soc. Trop. Med. Hyg..

[19] Bogarín G., Romero M., Rojas G., Lutsch C., Casadamont M., Lang J., Otero R., Gutiérrez J.M. (1999). Neutralization, by a monospecific *Bothrops lanceolatus* antivenom, of toxic activities induced by homologous and heterologous *Bothrops* snake venoms. Toxicon.

[20] Resiere D., Arias A.S., Villalta M., Rucavado A., Brouste Y., Cabié A., Névière R., Césaire R., Kallel H., Mégarbane B. (2018). Preclinical evaluation of the neutralizing ability of a monospecific antivenom for the treatment of envenoming by *Bothrops lanceolatus* in Martinique. Toxicon.

[21] WHO (2017). Guidelines for the Production, Control and Regulation of Snake Antivenom Immunoglobulins (Annex 5).

[22] Blessmann J., Hanlodsomphou S., Santisouk B., Krumkamp R., Kreuels B., Ismail A.K., Yong M.Y., Tan K.Y., Tan C.H. (2023). Experience of using expired lyophilized snake antivenom during a medical emergency situation in Lao People’s Democratic Republic A possible untapped resource to tackle antivenom shortage in Southeast Asia. Trop. Med. Int. Health.

[23] O’Leary M.A., Kornhauser R.S., Hodgson W.C., Isbister G.K. (2009). An examination of the activity of expired and mistreated commercial Australian antivenoms. Trans. R. Soc. Trop. Med. Hyg..

[24] Sanchez E.E., Migl C., Suntravat M., Rodriguez-Acosta A., Galan J.A., Salazar E. (2019). The neutralization efficacy of expired polyvalent antivenoms: An alternative option. Toxicon.

[25] Chippaux J.P., Williams V., White J. (1991). Snake venom variability: Methods of study, results and interpretation. Toxicon.

[26] Casewell N.R., Wagstaff S.C., Wüster W., Cook D.A.N., Bolton F.M.S., King S.I., Pla D., Sanz L., Calvete J.J., Harrison R.A. (2014). Medically important differences in snake venom composition are dictated by distinct postgenomic mechanisms. Proc. Natl. Acad. Sci. USA.

[27] Freitas-De-Sousa L.A., Nachtigall P.G., Portes-Junior J.A., Holding M.L., Nystrom G.S., Ellsworth S.A., Guimarães N.C., Tioyama E., Ortiz F., Silva B.R. (2020). Size matters: An evaluation of the molecular basis of ontogenetic modifications in the composition of *Bothrops jararacussu* snake venom. Toxins.

[28] Sousa L.F., Holding M.L., Del-Rei T.H.M., Rocha M.M.T., Mourão R.H.V., Chalkidis H.M., Prezoto B., Gibbs H.L., Moura-Da-Silva A.M. (2021). Individual variability in *Bothrops atrox* snakes collected from different habitats in the Brazilian Amazon: New findings on venom composition and functionality. Toxins.

[29] Bourke L.A., Zdenek C.N., Neri-Castro E., Bénard-Valle M., Alagón A., Gutiérrez J.M., Sanchez E.F., Aldridge M., Fry B.G. (2021). Pan-American lancehead pit-vipers: Coagulotoxic venom effects and antivenom neutralisation of *Bothrops asper* and *B. atrox* geographical variants. Toxins.

[30] Alsolaiss J., Alomran N., Hawkins L., Casewell N.R. (2022). Commercial antivenoms exert broad paraspecific immunological binding and in vitro inhibition of medically important *Bothrops* pit viper venoms. Toxins.

[31] León G., Herrera M., Segura Á., Villalta M., Vargas M., Gutiérrez J.M. (2013). Pathogenic mechanisms underlying adverse reactions induced by intravenous administration of snake antivenoms. Toxicon.

